# Experience of Robotic Partial Nephrectomy for Localized Renal Tumors: Functional and Oncologic Outcomes

**DOI:** 10.14740/wjon2791

**Published:** 2026-06-17

**Authors:** Tuan Thanh Nguyen, Khac Chuan Hoang, Trong Tri Tran, Le Quy Van Dinh, Xuan Thai Ngo

**Affiliations:** a University of Medicine and Pharmacy at Ho Chi Minh City, Vietnam; b Department of Urology, Cho Ray Hospital, Ho Chi Minh City, Vietnam

**Keywords:** Renal cell carcinoma, Robot-assisted partial nephrectomy, Oncologic outcomes, Trifecta, Renal tumor

## Abstract

**Background:**

Evidence comparing outcomes of robot-assisted partial nephrectomy (RAPN) between small and larger malignant renal tumors remains limited, particularly in real-world single-center practice. We aimed to compare perioperative, renal functional, and oncologic outcomes of RAPN according to tumor size and to evaluate predictors of trifecta achievement.

**Methods:**

We conducted a retrospective single-center cohort study of 74 patients undergoing transperitoneal RAPN for malignant renal tumors, including 42 patients with T1a tumors (≤ 4 cm) and 32 with tumors > 4 cm. Baseline characteristics, perioperative outcomes, renal functional outcomes, and oncologic outcomes were compared between groups. Trifecta was assessed using an exploratory parsimonious multivariable logistic regression model including tumor size group, RENAL complexity, hilar anatomy complexity, and age, given the limited sample size and event counts.

**Results:**

Compared with the T1a group, patients with tumors > 4 cm had significantly higher RENAL scores and a greater proportion of highly complex tumors. Operative time was longer (252.1 vs. 226.3 min, P = 0.030), and hospital stay was longer (median 6 vs. 5 days, P = 0.040). No statistically significant differences were observed in warm ischemia time, estimated blood loss, complication rates, positive surgical margin rates, or trifecta achievement (43.8% vs. 50.0%, P = 0.765), although the study may have been underpowered to detect clinically meaningful differences. Absolute estimated glomerular filtration rate (eGFR) was lower in the > 4 cm group at 3, 6, and 12 months, but eGFR preservation percentages and chronic kidney disease (CKD) upstaging rates did not differ significantly. On exploratory multivariable analysis, tumor size > 4 cm was not independently associated with trifecta achievement (odds ratio (OR) 0.80, 95% confidence interval (CI) 0.28–2.26, P = 0.673), and no statistically significant independent predictor was identified.

**Conclusions:**

RAPN for tumors > 4 cm was associated with greater anatomical complexity and modestly increased operative burden. No statistically significant differences were observed in ischemic, complication, oncologic, or relative renal functional outcomes, although these comparisons should be interpreted cautiously because the study may have been underpowered for uncommon events. These findings support the feasibility of RAPN for selected larger malignant renal tumors in a real-world single-center setting.

## Introduction

Partial nephrectomy is the standard nephron-sparing treatment for clinically localized renal tumors because it provides oncologic control comparable to radical nephrectomy while better preserving renal function [1]. In this setting, robot-assisted partial nephrectomy (RAPN) has become a preferred minimally invasive approach due to its three-dimensional visualization, improved dexterity, and greater operative precision, which help overcome several technical limitations of conventional laparoscopy [2–4].

As RAPN aims to balance cancer control, perioperative safety, and renal functional preservation, composite surgical quality metrics have gained increasing relevance. Among these, the trifecta—commonly defined as negative surgical margins, limited warm ischemia time (WIT), and the absence of significant perioperative complications—is widely used to reflect the quality of nephron-sparing surgery [5]. Tumor complexity remains a key determinant of RAPN difficulty, and nephrometry systems such as RENAL are routinely used to quantify anatomic features associated with more challenging resection and reconstruction [4].

Tumor size is one of the most clinically relevant markers of complexity. In the literature, T1a tumors are defined as measuring ≤ 4 cm, whereas T1b tumors measure > 4 cm and up to 7 cm [3]. Although RAPN has increasingly been applied to larger renal masses, tumors > 4 cm are generally associated with longer operative time, greater blood loss, and prolonged WIT compared with smaller tumors [6]. At the same time, RAPN for larger tumors appears feasible and oncologically safe in experienced hands [6]. However, direct comparisons stratified by tumor size remain limited, particularly when perioperative, renal functional, and oncologic outcomes are evaluated together and when predictors of trifecta achievement are specifically examined [7, 8].

This gap is especially relevant in Asia and Southeast Asia, where regional RAPN data remain limited. A recent Malaysian study emphasized the scarcity of Asian evidence for complex renal tumors, while a contemporary review of robotic urologic surgery in Southeast Asia highlighted substantial heterogeneity in robotic adoption, training, and institutional experience across the region [4, 9]. Therefore, this study aimed to compare the perioperative, renal functional, and oncologic outcomes of RAPN for malignant renal tumors according to tumor size (T1a, ≤ 4 cm, versus > 4 cm), and to identify clinical and surgical predictors associated with trifecta achievement in a retrospective single-center cohort.

## Materials and Methods

### Study design and setting

This study was conducted as a retrospective single-center cohort analysis at a tertiary referral institution. Consecutive patients who underwent transperitoneal RAPN for malignant renal tumors between January 2018 and December 2024 were screened for eligibility. Although the present analysis was retrospective, perioperative care and postoperative surveillance were performed according to standardized institutional practice during the study period.

This study was conducted in accordance with the ethical standards of the institutional research committee and with the 1964 Declaration of Helsinki and its later amendments, and reported following the STROBE recommendations for observational studies [10]. Ethical approval was obtained from the Institutional Review Board of the University of Medicine and Pharmacy at Ho Chi Minh City (Approval No. 1108/HDDD-DHYD, November 13, 2023).

### Patient selection

Eligible patients were adults who underwent transperitoneal RAPN for localized renal tumors during the study period. Patients were excluded if key clinical or perioperative variables were unavailable, or if the lesion represented an infectious/cystic process not eligible for the study cohort. After application of the predefined inclusion and exclusion criteria, the final analytic cohort comprised consecutive eligible patients included in the study database. For the main comparative analysis, patients were stratified according to tumor size into T1a (≤ 4 cm) and > 4 cm groups based on preoperative tumor diameter.

### Data collection and variable definitions

Baseline variables included age, sex, body mass index, and relevant comorbidity data. Tumor anatomical characteristics were assessed on preoperative contrast-enhanced imaging and summarized using the RENAL nephrometry scoring system [11]. Tumor complexity was categorized as low (4–6), moderate (7–9), or high (10–12). Hilar involvement was recorded as a binary anatomical feature.

**Figure 1:**
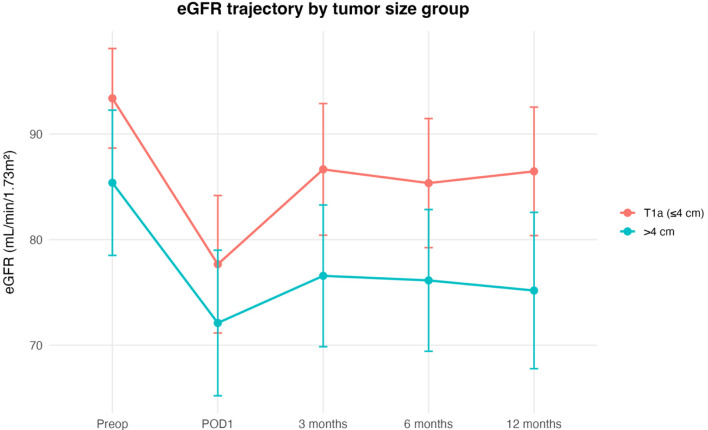
Renal function trajectory according to tumor size group after robot-assisted partial nephrectomy. Mean estimated glomerular filtration rate (eGFR) over time in patients undergoing robot-assisted partial nephrectomy, stratified by tumor size group: T1a (≤ 4 cm) and > 4 cm. Error bars indicate 95% confidence intervals. eGFR is expressed in mL/min/1.73 m^2^. POD1: postoperative day 1.

Perioperative variables included operative time, console time, WIT, estimated blood loss, and intraoperative events. Postoperative complications occurring within 30 days were graded according to the Clavien–Dindo classification [12]. Renal functional assessment was based on serum creatinine and estimated glomerular filtration rate (eGFR), calculated using the Chronic Kidney Disease Epidemiology Collaboration (CKD-EPI) equation, with measurements collected preoperatively and during routine postoperative follow-up [13]. Complete serum creatinine data were available for all patients at baseline and at 3, 6, and 12 months postoperatively. Therefore, the number of patients with available renal function data was identical across all time points shown in Figure 1.

For functional analyses, postoperative renal outcomes were assessed longitudinally at predefined follow-up time points according to data availability in the institutional database. Derived renal functional endpoints included postoperative eGFR preservation and chronic kidney disease (CKD) stage migration or upstaging, where applicable. Oncologic variables included surgical margin status and follow-up recurrence-related outcomes when available.

### Outcomes

The study was designed to evaluate outcomes across tumor size strata. The primary outcome for comparative analysis was trifecta achievement, defined as negative surgical margins, WIT ≤ 25 min, and absence of major postoperative complications. Major complications were defined as Clavien–Dindo grade ≥ 3.

Secondary outcomes included perioperative parameters such as operative time, console time, estimated blood loss, length of hospital stay, postoperative complications, and positive surgical margins. Additional secondary endpoints included postoperative renal functional outcomes, including eGFR preservation and CKD upstaging, as well as available oncologic follow-up outcomes.

### Surgical technique

All procedures were performed using a standardized transperitoneal RAPN technique with the da Vinci Si robotic platform (Intuitive Surgical, Sunnyvale, CA, USA) [14]. The robotic program at our institution was initiated in 2018, following several proctored cases in late 2017 under the supervision of an international expert. Three surgeons performed the vast majority of RAPN procedures in this cohort, accounting for approximately 90% of cases. These surgeons had extensive prior experience in laparoscopic renal surgery before adopting RAPN. Intraoperative ultrasound was not used in any case. Technical details were adapted to tumor anatomy and intraoperative findings at the discretion of the operating surgeon while maintaining the same overall transperitoneal robotic approach throughout the cohort.

### Statistical analysis

Continuous variables are presented as mean ± standard deviation or median with interquartile range, as appropriate according to data distribution. Categorical variables are summarized as frequencies and percentages.

Baseline characteristics and perioperative, functional, and oncologic outcomes were compared between the T1a (≤ 4 cm) and > 4 cm groups. For continuous variables, between-group comparisons were performed using the Student’s *t*-test or Mann–Whitney U test, as appropriate. For categorical variables, the Chi-square test or Fisher’s exact test was used.

To identify factors associated with trifecta achievement, univariable logistic regression analyses were first performed for clinically relevant demographic, tumor, and perioperative variables. Variables considered clinically important or statistically associated in univariable analysis were then entered into a multivariable logistic regression model constructed in a parsimonious manner to avoid model overfitting. Effect estimates are reported as odds ratios (ORs) with 95% confidence intervals (CIs).

All statistical analyses were performed using R (R Foundation for Statistical Computing, Vienna, Austria). Given the limited number of trifecta events and the rarity of several outcomes, the regression analysis was considered exploratory and parsimonious to reduce the risk of model overfitting. All tests were two-sided, and a P value < 0.05 was considered statistically significant.

## Results

### Study cohort

A total of 74 patients who underwent transperitoneal RAPN for localized renal tumors were included in the final analysis, including 42 patients with T1a tumors (≤ 4 cm) and 32 patients with tumors > 4 cm.

### Baseline characteristics

Baseline patient and tumor characteristics are summarized in Table 1. The two groups were generally comparable with respect to age, sex, body mass index, hypertension, diabetes mellitus, American Society of Anesthesiologists physical status classification (ASA) status, Eastern Cooperative Oncology Group (ECOG) performance status, Charlson Comorbidity Index, tumor laterality, complex hilar anatomy, preoperative hemoglobin, and preoperative eGFR.

**Table 1:** Baseline Patient and Tumor Characteristics According to Tumor Size Group

Variable	T1a (≤ 4 cm) (n = 42)	> 4 cm (n = 32)	P value
Age (years), mean (SD)	52.86 (11.64)	57.19 (12.21)	0.116
Male patients, n (%)	25 (59.5%)	21 (65.6%)	0.769
BMI, mean (SD)	25.64 (3.97)	24.12 (3.90)	0.060
HTN, n (%)	22 (52.4%)	13 (40.6%)	0.442
DM, n (%)	7 (16.7%)	5 (15.6%)	1.000
ASA status, n (%)			0.455
1	3 (7.1%)	5 (15.6%)	
2	27 (64.3%)	17 (53.1%)	
3	12 (28.6%)	10 (31.2%)	
ECOG performance status, n (%)			0.326
0	34 (81.0%)	23 (71.9%)	
1	7 (16.7%)	9 (28.1%)	
2	1 (2.4%)	0 (0.0%)	
CCI age-adjusted, median (IQR)	3 (2–4)	4 (2–4)	0.113
Left-sided tumor, n (%)	22 (52.4%)	18 (56.2%)	0.924
cT, n (%)			< 0.001
cT1a	42 (100.0%)	0 (0.0%)	
cT1b	0 (0.0%)	30 (93.8%)	
cT2a	0 (0.0%)	2 (6.2%)	
Tumor size (cm), median (IQR)	3.10 (2.78–3.50)	5.00 (4.50–6.00)	< 0.001
Hilar tumor, n (%)	3 (7.1%)	7 (21.9%)	0.090
RENAL score, median (IQR)	7 (5–8)	9 (8–10)	< 0.001
RENAL score category, n (%)			< 0.001
Low complexity (4–6)	17 (40.5%)	2 (6.2%)	
Moderate complexity (7–9)	21 (50.0%)	18 (56.2%)	
High complexity (10–12)	4 (9.5%)	12 (37.5%)	
Complex hilar anatomy (> 1 renal vein or > 1 renal artery), n (%)	6 (14.3%)	6 (18.8%)	0.843
Preoperative hemoglobin (g/dL), mean (SD)	142.21 (14.97)	140.19 (16.83)	0.814
Preoperative GFR (mL/min/1.73 m^2^), mean (SD)	93.38 (15.60)	85.38 (19.84)	0.057

ASA: American Society of Anesthesiologists physical status classification; BMI: body mass index; CCI: Charlson Comorbidity Index; cT: clinical tumor stage; DM: diabetes mellitus; ECOG: Eastern Cooperative Oncology Group; GFR: glomerular filtration rate; HTN: hypertension; IQR: interquartile range; RENAL: Radius, Exophytic/endophytic properties, Nearness to the collecting system, Anterior/posterior location, and Location relative to polar lines.

As expected, the > 4 cm group had more advanced clinical stage distribution, larger tumor size, and greater anatomical complexity. Specifically, this group showed a higher median tumor diameter (5.0 vs. 3.1 cm, P < 0.001), a higher median RENAL score (9 vs. 7, P < 0.001), and a less favorable RENAL complexity distribution, with a greater proportion of highly complex tumors (37.5% vs. 9.5%, P < 0.001). Hilar tumors were also more frequent in the > 4 cm group, although this difference did not reach statistical significance (21.9% vs. 7.1%, P = 0.09).

### Perioperative and postoperative outcomes

Perioperative and postoperative outcomes are presented in Table 2, whereas renal functional and oncologic follow-up outcomes are summarized in Table 3. Compared with the T1a group, patients with tumors > 4 cm had a significantly longer operative time (252.1 ± 39.2 vs. 226.3 ± 55.2 min, P = 0.030) and a longer hospital stay (median 6 vs. 5 days, P = 0.040). In contrast, console time, WIT, estimated blood loss, postoperative pain score, opioid requirement, transfusion rate, conversion to open surgery, and conversion to radical nephrectomy were similar between groups.

**Table 2:** Perioperative and Postoperative Outcomes According to Tumor Size Group

Variable	T1a (≤ 4 cm) (n = 42)	> 4 cm (n = 32)	P value
Postoperative hemoglobin (g/L), mean (SD)	126 (15.2)	119.7 (16.4)	0.153
Operation time (min), mean (SD)	226.33 (55.21)	252.06 (39.16)	0.030*
Console time (min), mean (SD)	164.18 (41.19)	170.50 (52.26)	0.564
WIT (min), mean (SD)	27.00 (9.43)	27.44 (9.73)	0.611
WIT ≤ 25 min, n (%)	21 (50.0%)	15 (46.9%)	0.975
EBL (mL), mean (SD)	80.4 (75.9)	73.6 (44)	0.499
Transfusion required, n (%)	0 (0.0%)	2 (6.2%)	0.184
Conversion to open surgery, n (%)	0 (0.0%)	0 (0.0%)	1.000
Conversion to radical nephrectomy, n (%)	0 (0.0%)	0 (0.0%)	1.000
Hemoglobin drop (g/L), mean (SD)	16.7 (9)	20.5 (9.9)	0.065
Pain score (0–10), median (IQR)	4.00 (3.00–4.00)	3.50 (3.00–5.00)	0.853
Opioid use, n (%)	16 (38.1%)	18 (56.2%)	0.188
Hospital stay (days), median (IQR)	5.00 (4.00–6.00)	6.00 (5.00–7.00)	0.040*
Positive surgical margin, n (%)	1 (2.4%)	0 (0.0%)	1.000
Postoperative complications, n (%)	3 (7.1%)	6 (18.8%)	0.163
Major postoperative complication (Clavien–Dindo ≥ 3), n (%)	0 (0.0%)	1 (3.1%)	0.432
Trifecta achieved, n (%)	21 (50.0%)	14 (43.8%)	0.765

*Significant difference. EBL: estimated blood loss; IQR: interquartile range; SD: standard deviation; WIT: warm ischemia time.

**Table 3:** Renal Functional and Oncologic Follow-Up Outcomes According to Tumor Size Group

Variable	T1a (≤ 4 cm) (n = 42)	> 4 cm (n = 32)	P value
POD1 GFR (mL/min/1.73 m^2^), mean (SD)	75.37 (20.43)	71.73 (20.10)	0.468
3-month GFR (mL/min/1.73 m^2^), mean (SD)	86.65 (20.61)	76.57 (19.36)	0.034*
6-month GFR (mL/min/1.73 m^2^), mean (SD)	85.35 (20.21)	76.14 (19.37)	0.033*
12-month GFR (mL/min/1.73 m^2^), mean (SD)	86.46 (20.11)	75.18 (21.36)	0.012*
eGFR preservation at 3 months (%), mean (SD)	93.07 (18.34)	91.32 (17.82)	0.600
eGFR preservation at 6 months (%), mean (SD)	91.69 (18.04)	91.32 (19.89)	0.836
eGFR preservation at 12 months (%), mean (SD)	92.68 (17.47)	88.15 (16.45)	0.280
CKD upstage at 12 months, n (%)	9 (21.4%)	11 (34.4%)	0.328
Pathology, n (%)			0.321
Clear cell RCC	28 (66.7%)	19 (59.4%)	
Papillary RCC	4 (9.5%)	8 (25.0%)	
Other pathology	10 (23.8%)	5 (15.6%)	
Follow-up time (months), median (IQR)	41.5 (18.3–64.8)	52.5 (19.5–77)	0.781
Recurrence, n (%)	0 (0.0%)	1 (3.1%)	0.432

*Significant difference. CKD: chronic kidney disease; eGFR: estimated glomerular filtration rate; IQR: interquartile range; POD1: postoperative day 1; SD: standard deviation.

WIT was comparable between the two groups (27.4 ± 9.7 vs. 27.0 ± 9.4 min, P = 0.611), and the proportion of patients achieving WIT ≤ 25 min did not differ significantly (46.9% vs. 50.0%, P = 0.975). Estimated blood loss was also similar (73.6 ± 44.0 vs. 80.4 ± 75.9 mL, P = 0.499). Although postoperative hemoglobin drop tended to be greater in the > 4 cm group, the difference was not statistically significant (20.5 ± 9.9 vs. 16.7 ± 9.0 g/L, P = 0.065).

Postoperative complications occurred in 18.8% of patients in the > 4 cm group and 7.1% in the T1a group, but this difference was not statistically significant (P = 0.163). Major postoperative complications were uncommon in both groups (0% vs. 3.1%, P = 0.432). Positive surgical margins were rare, with only one event observed in the T1a group (2.4% vs. 0%, P = 1.000). Trifecta achievement was comparable between groups (50.0% vs. 43.8%, P = 0.765).

### Renal functional outcomes

Early postoperative renal function was similar between groups. Postoperative day 1 eGFR did not differ significantly between the T1a and > 4 cm groups (75.4 ± 20.4 vs. 71.7 ± 20.1 mL/min/1.73 m^2^, P = 0.468). However, during follow-up, the > 4 cm group showed lower absolute eGFR values at 3 months (76.6 vs. 86.7, P = 0.034), 6 months (76.1 vs. 85.4, P = 0.033), and 12 months (75.2 vs. 86.5, P = 0.012).

Despite these differences in absolute eGFR, the degree of relative renal function preservation was comparable between groups. Mean eGFR preservation at 3, 6, and 12 months did not significantly differ, and the rate of CKD upstaging at 12 months was also similar (34.4% vs. 21.4%, P = 0.328). The renal function trajectory is illustrated in Figure 1, which shows an immediate postoperative decline in eGFR in both groups followed by partial recovery over time, with consistently lower absolute eGFR values in the > 4 cm group.

### Pathologic and oncologic outcomes

Pathologic subtype distribution did not differ significantly between groups (P = 0.321). Clear cell renal cell carcinoma was the most common histology in both cohorts. Median follow-up duration was comparable (52.5 vs. 41.5 months, P = 0.781). Recurrence was rare, with one event observed in the > 4 cm group and none in the T1a group (3.1% vs. 0%, P = 0.432).

### Predictors of trifecta achievement

**Figure 2:**
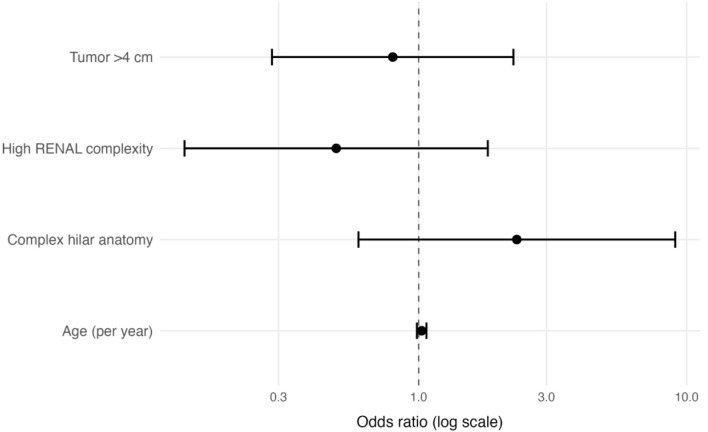
Forest plot of multivariable predictors of trifecta achievement. Forest plot showing odds ratios and 95% confidence intervals from the multivariable logistic regression model for predictors of trifecta achievement after robot-assisted partial nephrectomy. The model included tumor size group, RENAL complexity group, hilar anatomy complexity, and age. The dashed vertical line indicates an odds ratio of 1.0. Odds ratios are displayed on a logarithmic scale.

Univariable and multivariable logistic regression analyses for predictors of trifecta achievement are shown in Table 4, with the multivariable model visualized in Figure 2. In univariable analysis, tumor size > 4 cm, high RENAL complexity, complex hilar anatomy, and age were not significantly associated with trifecta achievement.

**Table 4:** Univariable and Multivariable Logistic Regression Analyses for Predictors of Trifecta Achievement

Variable	Univariable OR (95% CI)	Univariable P	Multivariable OR (95% CI)	Multivariable P
Tumor > 4 cm (ref: T1a ≤ 4 cm)	0.78 (0.31–1.96)	0.594	0.80 (0.28–2.26)	0.673
High RENAL complexity (ref: low–moderate)	0.42 (0.12–1.32)	0.153	0.49 (0.12–1.76)	0.286
Complex hilar anatomy (ref: simple)	2.59 (0.74–10.56)	0.151	2.32 (0.62–9.99)	0.225
Age (per year)	1.02 (0.98–1.06)	0.337	1.03 (0.99–1.07)	0.221

The multivariable model was intentionally kept parsimonious because of the small sample size. On multivariable logistic regression analysis, predictors of trifecta achievement included tumor size > 4 cm (OR = 0.80, 95% CI 0.28–2.26, P = 0.673); high RENAL complexity (OR = 0.49, 95% CI 0.12–1.76, P = 0.286); complex hilar anatomy (OR = 2.32, 95% CI 0.62–9.99, P = 0.225); older age (OR = 1.03, 95% CI 0.99–1.07, P = 0.221). CI: confidence interval; OR: odds ratio.

In the parsimonious multivariable model, no variable was independently associated with trifecta achievement. Tumor size > 4 cm showed an adjusted OR of 0.80 (95% CI 0.28–2.26, P = 0.673), high RENAL complexity showed an OR of 0.49 (95% CI 0.12–1.76, P = 0.286), complex hilar anatomy showed an OR of 2.32 (95% CI 0.62–9.99, P = 0.225), and age showed an OR of 1.03 per year (95% CI 0.99–1.07, P = 0.221). Overall, the forest plot demonstrates that all CIs crossed unity, indicating the absence of a statistically significant independent predictor of trifecta achievement in this cohort.

## Discussion

In this retrospective single-center cohort of patients undergoing transperitoneal RAPN for malignant renal tumors, tumors > 4 cm were associated with greater anatomical complexity, longer operative time, and longer hospital stay than T1a tumors. No statistically significant differences were observed in WIT, estimated blood loss, complication rates, positive surgical margin rates, or trifecta achievement between groups; however, these comparisons should be interpreted cautiously because uncommon events were rare and the study may have been underpowered to detect clinically meaningful differences. In addition, although the > 4 cm group had lower absolute postoperative eGFR values during follow-up, the relative degree of renal function preservation and the rate of CKD upstaging were not significantly different. Finally, no statistically significant independent predictor of trifecta achievement was identified in the exploratory parsimonious multivariable model.

The finding that larger tumors were accompanied by higher anatomical complexity is expected and consistent with prior RAPN literature. In comparative series, tumors > 4 cm or T1b lesions generally carry higher nephrometry scores and more challenging spatial relationships to the collecting system and renal hilum, which translate into more demanding tumor excision and renorrhaphy [6, 15]. This likely explains why operative time was longer in our > 4 cm group. A modest prolongation in hospital stay may also reflect the greater extent of resection and the need for more cautious postoperative observation after surgery for larger tumors. Importantly, these differences appear to reflect technical complexity rather than overt deterioration in perioperative safety.

Despite the higher complexity of tumors > 4 cm, we did not observe statistically significant differences in WIT or trifecta achievement. This finding should not be interpreted as evidence of equivalence, because the cohort was modest in size and events relevant to trifecta were infrequent. In our series, trifecta rates were influenced largely by the warm ischemia threshold itself: mean WIT was approximately 27 min in both groups, and only about half of patients achieved WIT ≤ 25 min, whereas positive surgical margins and major complications were rare. Accordingly, the relatively low trifecta rates appear to reflect the stringency of the WIT criterion more than frequent failures in margin control or severe perioperative morbidity. The enhanced visualization, instrument articulation, and suturing control of RAPN may still help surgeons maintain acceptable ischemia times in anatomically challenging cases [1, 16, 17]. Our findings are broadly in line with the study by Sharma et al, in which trifecta rates remained comparable between T1a and T1b tumors despite longer WITs in the larger-tumor cohort [8]. At the same time, our results differ from broader multicenter experiences in which larger tumor size was associated with worse trifecta outcomes, suggesting that the effect of tumor size may be modified by institutional experience, case selection, and standardization of operative technique [18–20].

The median hospital stay in our cohort (5–6 days) was longer than that in many contemporary RAPN reports and likely reflects institutional discharge practices and healthcare-system factors rather than surgical morbidity alone. Discharge at our center is generally based on clinical stabilization, laboratory reassessment, pain control, mobilization, and drain/postoperative monitoring when indicated. Notably, postoperative length of stay after partial nephrectomy remains heterogeneous across institutions [21]. In a contemporary Japanese series, Hatayama et al reported a median postoperative hospital stay of 7 days after both laparoscopic and RAPN. In addition, Walach et al evaluated length of stay > 7 days as a clinically relevant postoperative endpoint in patients undergoing partial nephrectomy [21–23].

The renal functional findings also warrant careful interpretation. In our cohort, patients with tumors > 4 cm had lower absolute eGFR values at 3, 6, and 12 months, yet the percentage of eGFR preservation did not significantly differ between groups. Importantly, preoperative eGFR was also lower in the > 4 cm group and was near the threshold for statistical significance, suggesting that the lower absolute postoperative eGFR values may partly reflect baseline imbalance rather than a purely surgery-related decrement. This pattern suggests that treatment of larger and more complex lesions may still preserve renal function proportionally even when postoperative absolute values remain lower. Prior studies have similarly shown that although larger or more complex tumors may be associated with more challenging surgery, the relative decline in renal function is not always substantially worse, particularly when an acceptable amount of parenchyma is preserved [6, 24]. More broadly, functional recovery after RAPN is determined not only by ischemia time, but also by the amount and quality of the residual parenchyma, emphasizing that tissue preservation remains central to nephron-sparing success [25, 26].

Our oncologic findings were reassuring but should be interpreted cautiously. Positive surgical margins were rare and recurrence was uncommon, supporting favorable early oncologic outcomes in both tumor-size groups. However, only one recurrence, one major complication, and one positive margin were observed in the entire cohort, and these sparse events make between-group comparisons of oncologic safety, complication risk, and predictors of trifecta inherently unstable. Accordingly, the absence of statistically significant differences should not be overstated. In parallel, the absence of independent predictors of trifecta achievement in our multivariable model likely reflects the limited sample size and the relatively small number of events, rather than definitive absence of association. Previous studies suggest that surgeon experience, program maturity, and granular anatomical factors may outweigh tumor diameter alone when predicting RAPN quality metrics [8, 19, 27]. Accordingly, our negative multivariable findings are best interpreted as hypothesis-generating rather than conclusive.

These data should also be interpreted within the context of RAPN implementation in Asia and in developing robotic programs. Prior work has emphasized that evidence from Asia, and particularly from Southeast Asia, remains more limited than that from Western high-volume centers [4]. At the same time, robotic surgery uptake across the region is heterogeneous, shaped by differences in infrastructure, access, training pathways, and institutional experience [4, 9]. In that context, real-world single-center data remain valuable because they reflect how RAPN performs outside highly selected benchmark centers. Our findings suggest that acceptable perioperative, functional, and early oncologic outcomes can be achieved even when larger tumors are treated in an emerging robotic environment, provided that case selection, perioperative protocols, and surgical technique are applied consistently. This may be particularly relevant for newer centers in Asia seeking to expand nephron-sparing surgery beyond small renal masses.

This study has several limitations. First, its retrospective single-center design introduces the possibility of selection bias and unmeasured confounding. Second, the sample size was modest, especially for the > 4 cm cohort and for exploratory multivariable modeling of trifecta achievement, which may have limited statistical power. Third, several clinically important outcomes were rare, making comparisons of oncologic safety and major complications unstable. Fourth, although follow-up was sufficient for reporting early oncologic outcomes, it remains inadequate to draw firm conclusions regarding long-term recurrence or renal functional durability. Fifth, renal function was assessed using serum creatinine-based eGFR rather than split renal function or volumetric parenchymal assessment, which may better capture the true functional consequences of nephron-sparing surgery. Sixth, we did not collect granular surgeon-level variables such as individual RAPN experience, annual case volume, fellowship background, or learning-curve position, and we did not formally analyze program maturity; these factors may influence operative time, ischemia, complications, and trifecta achievement. Finally, our findings reflect the experience and protocols of a single institution and may not be fully generalizable to centers with different case mix, surgeon background, or stage of robotic program development.

### Conclusion

In summary, RAPN for tumors > 4 cm was associated with greater tumor complexity and modestly increased operative burden. No statistically significant differences were observed in WIT, complications, trifecta achievement, or relative renal functional preservation compared with T1a tumors, but these comparisons should be interpreted cautiously because the study was modest in size and uncommon adverse events were rare. The lower absolute postoperative eGFR values in the > 4 cm group should also be interpreted in the context of lower baseline preoperative renal function. Overall, our findings support the feasibility of RAPN for selected larger malignant renal tumors in a real-world single-center setting. Larger prospective studies with longer follow-up are needed to better define the functional and oncologic implications of tumor size and to clarify predictors of high-quality surgical outcomes after RAPN.

## Data Availability

De-identified datasets used and analyzed during the current study are available from the corresponding author upon reasonable request.

## Abbreviations

ASA: American Society of Anesthesiologists physical status classification
BMI: body mass index
CCI: Charlson Comorbidity Index
CI: confidence interval
CKD: chronic kidney disease
CKD-EPI: Chronic Kidney Disease Epidemiology Collaboration
cT: clinical tumor stage
DM: diabetes mellitus
ECOG: Eastern Cooperative Oncology Group
eGFR: estimated glomerular filtration rate
GFR: glomerular filtration rate
HTN: hypertension
IQR: interquartile range
OR: odds ratio
POD1: postoperative day 1
PN: partial nephrectomy
PSM: positive surgical margin
RAPN: robot-assisted partial nephrectomy
RCC: renal cell carcinoma
RENAL: Radius, Exophytic/endophytic properties, Nearness to the collecting system or sinus, Anterior/posterior location, and Location relative to the polar lines
SD: standard deviation
STROBE: Strengthening the Reporting of Observational Studies in Epidemiology
WIT: warm ischemia time
